# Mast Cell Dependent Vascular Changes Associated with an Acute Response to Cold Immersion in Primary Contact Urticaria

**DOI:** 10.1371/journal.pone.0056773

**Published:** 2013-02-22

**Authors:** Joseph Meyer, Alexander M. Gorbach, Wei-Min Liu, Nevenka Medic, Michael Young, Celeste Nelson, Sarah Arceo, Avanti Desai, Dean D. Metcalfe, Hirsh D. Komarow

**Affiliations:** 1 Infrared Imaging and Thermometry Unit, National Institute of Biomedical Imaging and Bioengineering, National Institutes of Health, Bethesda, Maryland, United States of America; 2 Laboratory of Allergic Diseases, National Institute of Allergy and Infectious Diseases, National Institutes of Health, Bethesda, Maryland, United States of America; 3 Clinical Research Directorate/CMRP, SAIC-Frederick, NCI Frederick, Frederick, Maryland, United States of America; King’s College London School of Medicine, United Kingdom

## Abstract

**Background:**

While a number of the consequences of mast cell degranulation within tissues have been documented including tissue-specific changes such as bronchospasm and the subsequent cellular infiltrate, there is little known about the immediate effects of mast cell degranulation on the associated vasculature, critical to understanding the evolution of mast cell dependent inflammation.

**Objective:**

To characterize the microcirculatory events that follow mast cell degranulation.

**Methodology/Principal Findings:**

Perturbations in dermal blood flow, temperature and skin color were analyzed using laser-speckle contrast imaging, infrared and polarized-light colorimetry following cold-hand immersion (CHI) challenge in patients with cold-induced urticaria compared to the response in healthy controls. Evidence for mast cell degranulation was established by documentation of serum histamine levels and the localized release of tryptase in post-challenge urticarial biopsies. Laser-speckle contrast imaging quantified the attenuated response to cold challenge in patients on cetirizine. We found that the histamine-associated vascular response accompanying mast cell degranulation is rapid and extensive. At the tissue level, it is characterized by a uniform pattern of increased blood flow, thermal warming, vasodilation, and recruitment of collateral circulation. These vascular responses are modified by the administration of an antihistamine.

**Conclusions/Significance:**

Monitoring the hemodynamic responses within tissues that are associated with mast cell degranulation provides additional insight into the evolution of the acute inflammatory response and offers a unique approach to assess the effectiveness of treatment intervention.

## Introduction

Mast cell activation and mediator release associated with allergic inflammation is correlated with a number of immediate physiologic changes within affected tissues including bronchospasm and mucus production in the lungs; abdominal pain associated with edema and diarrhea in the gastrointestinal tract and pruritic edema in the skin [1], [2], [3]. Microscopically mast cell activation is then followed by influx of inflammatory cells [4], [5], [6], [7]. However in spite of such observations, little is known about the direct immediate and critical effect of human mast cell degranulation on regional blood flow and the vasculature in the area of the evolving acute response.

In order to better understand these vascular responses in human tissues, we chose to initiate mast cell degranulation by a physical stimulus known to activate mast cells [8]. Mast cell degradation appears to be the feature that differentiates the consequences of cold exposure between normal individuals and those with cold urticaria [9], [10]. We therefore designed a clinical study to induce mast cell degranulation by cold challenge in patients with cold urticaria (CUrt). To determine the timing and character of vascular changes, we employed real time optical imaging. Mast cell degranulation was confirmed histologically by examining mast cells within tissue and by measuring histamine in venous flow passing through the site of cold challenge.

As will be shown, the vascular response associated with mast cell degranulation is rapid and extensive and is modified by the administration of an antihistamine, a proof of principle that the effect of anti-allergic compounds that impact the consequences of mast cell degranulation [11]may be monitored using this unique approach.

## Results

### Confirmation of Mast Cell Degranulation

**Figure 1:**
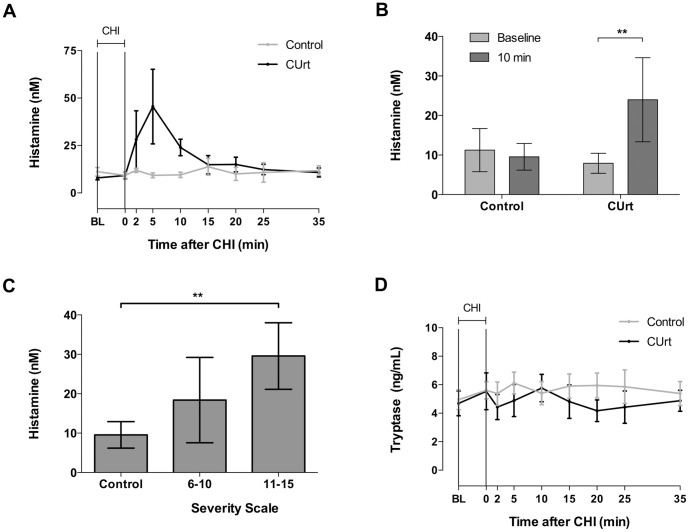
Histamine data and severity scale. Time profile of histamine release (A) for healthy controls and CUrt patients. Panel B shows the comparison of controls and CUrt patients at baseline and 10 minutes post-CHI for histamine. Panel C shows a comparison of normal control subjects and patients for histamine levels stratified by severity scale at 10 minutes post-CHI. Significance level, **p<0.01. No elevation in tryptase was detected in patients (D, p = 0.42, RM ANOVA) or controls (p = 0.48). In data not shown, no elevation in tryptase was detected through 120 minutes following CHI. Data shown as mean ± SEM. CHI challenge period is indicated with a horizontal bar in Panels A and D.

We first verified that our inducible procedures were accompanied by histamine release and that such evidence of mast cell activation correlated with the severity of the reactions. The mean group time profiles for serum histamine for healthy controls and patients with CUrt is shown in Figure 1A. Although there was variability amongst patients (as indicated by SEM), histamine peaked at 45.5 nM after 5 minutes of rewarming, as expected [12]. Histamine levels were then compared at baseline and 10 minutes following cold hand immersion (CHI) at the peak of the vascular response in order to evaluate the histamine peak related to severity of the induced reaction. At 10 minutes, the mean histamine concentration was significantly increased in patients (Figure 1B, p = 0.006), while that of controls remained unchanged (p = 0.53). Although there was expected variability amongst patients as indicated by SEM, serum histamine increased the most in the highest severity group after 10 minutes of rewarming (Figure 1C). Furthermore, there was significant correlation between histamine levels and disease severity (r = 0.79, p = 0.002, Figure S1) Thus, our challenge procedure triggered histamine release reflecting mast cell degranulation which related to the severity of the reaction.

**Figure 2:**
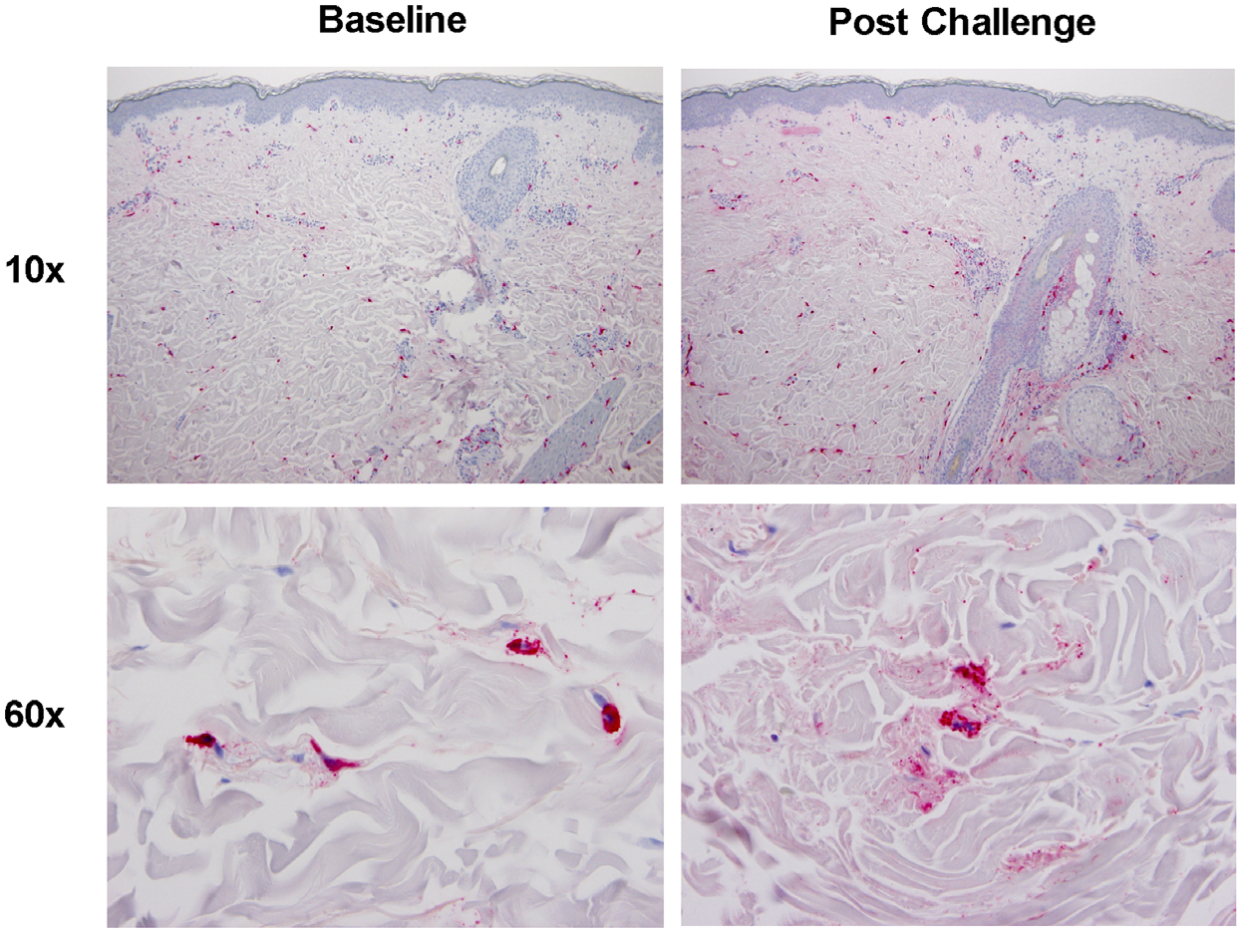
Tryptase-stained skin biopsy. Skin biopsy in CUrt patient stained for tryptase (red) at baseline at low (10×, upper panels) and high (60×, lower panels) magnification and at 15 minutes following cold stimulation time test (CSTT-see methods).

Serum tryptase did not increase over 35 minutes, consistent with previous reports [13] (Figure 1D, p = 0.24, ANOVA). However, compared to baseline, post challenge histological sections (n = 4) of dermis in a representative patient revealed increase in staining of tryptase diffusely within dermal tissues (Figure 2, compare left upper panel with right upper panel). Closer inspection of individual mast cells revealed both increase of tryptase staining in surrounding tissue and numerous extracellular mast cell granules (compare left lower panel with right lower panel) following cold challenge. Normal volunteer biopsies did not show increased staining of granular tryptase post challenge. These findings in association with the documentation of histamine release provide evidence of mast cell degranulation following CHI in affected subjects [14], [15].

### Real-time Imaging of Vascular Changes Associated with Mast Cell Degranulation

**Figure 3:**
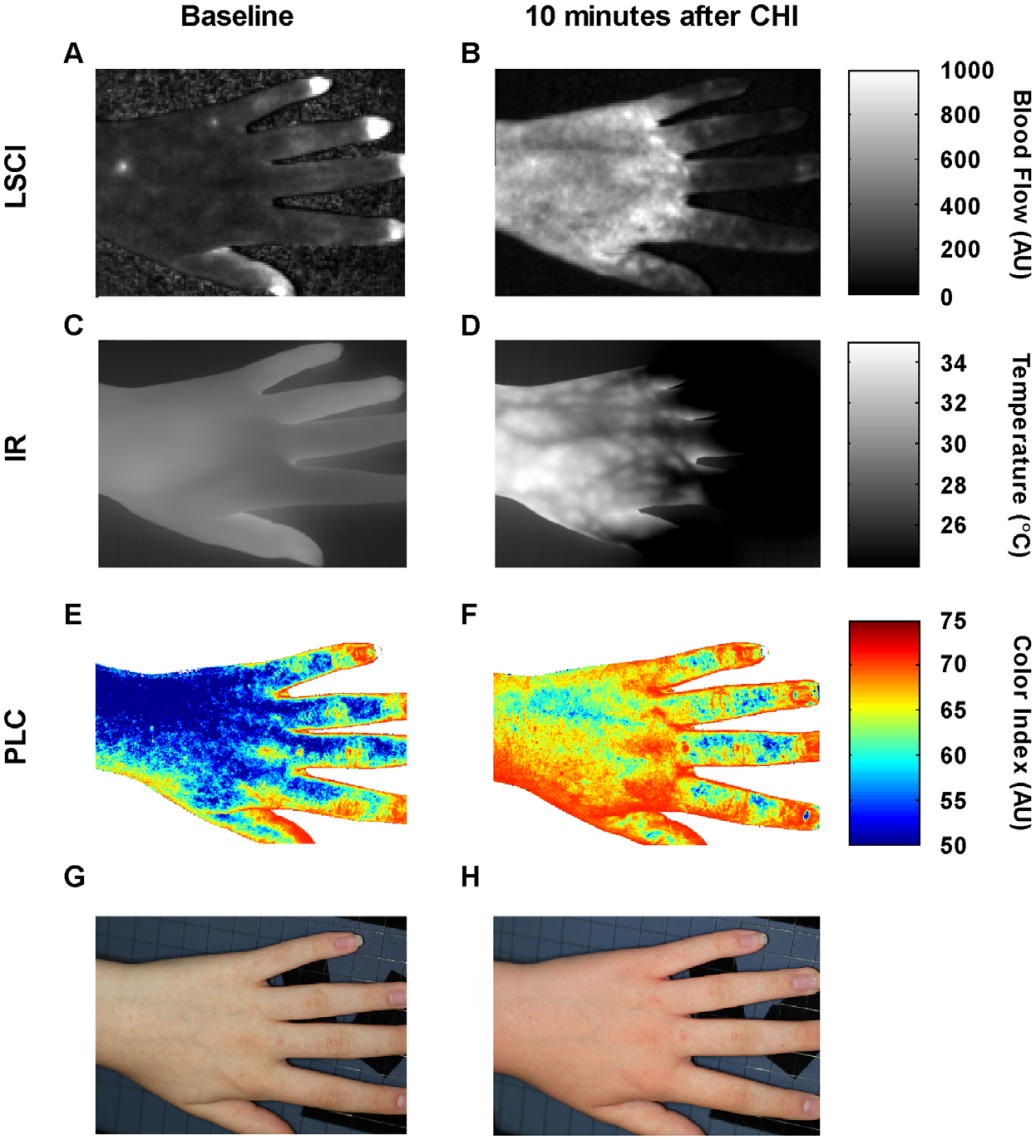
Blood flow, temperature, and skin color images of a representative CUrt subject. Images at baseline (A, C, E, and G) and at 10 minutes post-CHI (B, D, F, and H) for a CUrt subject (Table S1, Subject 6). Panel A and B, show blood flow images by LSCI; C and D, the temperature images by infrared (IR); E and F, the skin color images by polarized light colorimetry (PLC); and G and H, visible light photography. The blood flow image in A has been scaled up by a factor of 4 for visibility.

Real-time images were collected in all subjects before and after CHI using three cameras. Images of a representative CUrt subject at baseline and upon rewarming 10 minutes after CHI are shown in Figure 3. At baseline, blood flow (Figure 3A), temperature, (Figure 3C), color index (Figure 3E), and gross macroscopic changes (Figure 3G) are relatively homogeneous and of low intensity across the dorsum of the hand. Ten minutes post-CHI, at the peak of the vascular response dramatic changes are observed in the regional temperature and vascular flow. As can be seen in Figure 3B, superficial blood flow increased greatly in the hand relative to the fingers and multiple distinct patches of high tissue perfusion emerge. Similarly, in deeper tissue, IR detected increases in temperature, implicating involvement of small vascular beds that evolve to encompass nearby tissues but spare the digits (Figure 3D). These changes were associated with an increase in red blood cell concentration in superficial tissues (Figure 3F) which clearly paralleled the visual changes in skin color and tissue edema of both the hand and fingers (Figure 3H). Based on these observations, it appears that small superficial vessels dilate in response to cold challenge, thereby increasing superficial red cell concentration, but the underlying larger arterioles constrict, thereby reducing total blood flow in the fingers. For comparison, images of a control subject, which show minimal changes following CHI, are presented in supporting information (Figure S2). A real time infrared temperature video of the vascular changes that occur in a patient during CHI and the corresponding release of serum histamine can be viewed from the link in supporting information (Video S1). These results are consistent with but do not prove that mast cell degranulation within cutaneous tissues is the one inducer associated with alterations both in superficial blood flow and in deeper small vascular beds.

**Figure 4:**
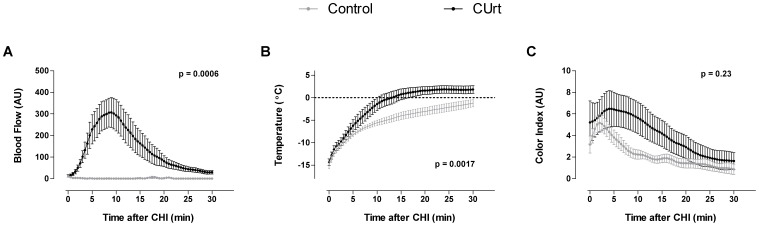
Imaging time profiles for healthy controls and CUrt subjects. Mean blow flow (A), temperature (B), and color index (C) are shown for healthy controls (gray) and patient with Curt (black). For summary data, mean baseline was subtracted from each individual time profile, and then the profiles were smoothed, down-sampled, and averaged based on subject groups. Significant differences between CUrt and control groups are seen for LSCI (A) and IR (B) imagers, but not PLC (C) as calculated by 2-way ANOVA. The dotted horizontal line in panel B represents baseline temperature.

**Figure 5:**
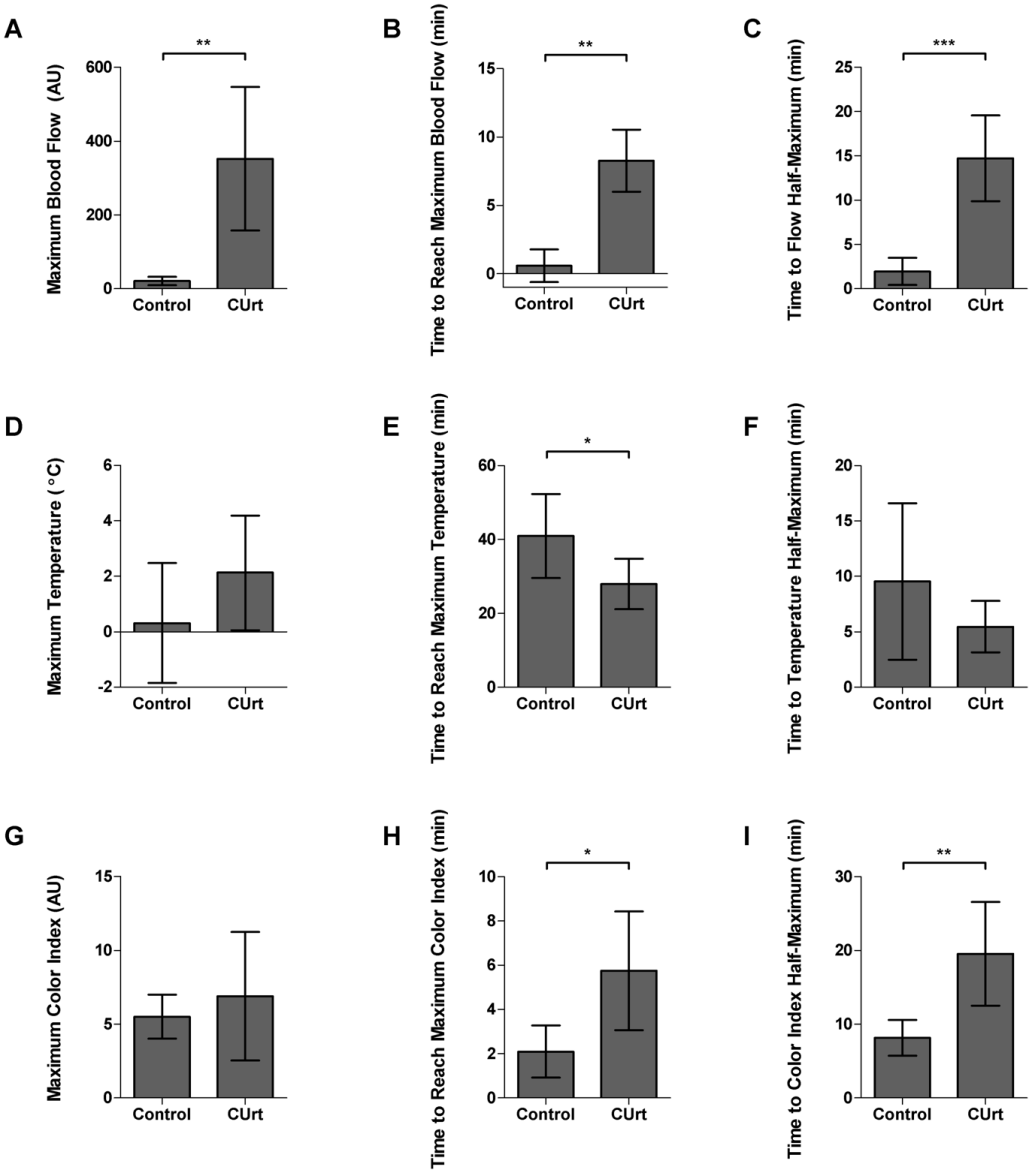
Maximum response marker: differences between healthy control and CUrt groups for blood flow, temperature, and skin color. Maximum value above baseline (A, D, and G) and the time it was reached (B, E, and H) were calculated for LSCI (A, B, C), IR (D, E, F), and PLC (G, H, I). Recovery time marker: differences between control and CUrt groups for blood flow, temperature, and skin color. Time of recovery to reach half of maximum for LSCI (G), IR (H), and PLC (I).

We then analyzed the temporal sequence in changes in blood flow associated with mast cell activation. Plotting the mean values versus time of rewarming after CHI (Figure 4) revealed significant differences in the temporal domain for blood flow (Figure 4A, p = 0.0006), temperature (Figure 4B, p = 0.0017), and color (Figure 4C, p = 0.23). In affected patients, the increase in blood flow was rapid, peaking at 7–9 minutes. Blood flow changes were persistent and did not return to approximate baseline for 25 minutes. Control subjects where there was no mast cell activation, had no change in blood flow (Figure 4A). Statistical analysis of blood flow is shown in Figure 5A, by unpaired t-test with Welch’s correction (p = 0.004) and Figure 5B (maximal blood flow), by rank-sum, p = 0.003. Patients also displayed more rapid tissue rewarming which somewhat exceeded baseline temperature at 15 through 30 minutes (Figures 4B, and 5D, maximum above baseline). The tissue temperature in controls more slowly returned to baseline (Figures 4B, 5E, time to maximum temperature). The mean values for maximal color index were not statistically significant between patients and controls (Figure 5G, p = 0.53), but the maximum was reached more slowly in those with CUrt (Figure 4C; Figure 5H, p = 0.018). The rate of recovery as reflected in the time required to return to half of maximum (Figures 5C, F and I) also revealed differences in subjects. Patients’ recovery was significantly slower than controls as assessed by blood flow (Figure 5C, p<0.001) and color (Figure 5I, 19.5 min vs. 8.2 min, p = 0.006), but not by temperature (Figure 5F, p = 0.21). This data is consistent with the conclusion that patients have an exaggerated response to cold as evidenced by a significant increase in blood flow which accelerates tissue rewarming and which is a consequence of mast cell degranulation.

**Figure 6:**
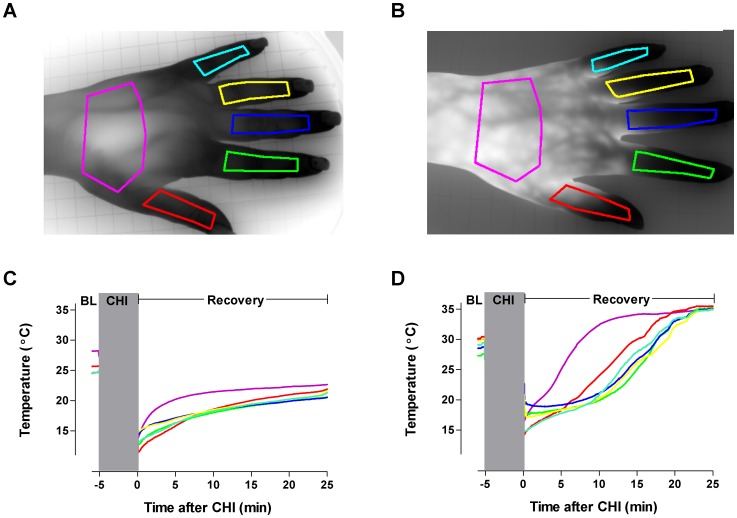
Comparison of the temperature recovery of a healthy control and CUrt subject. Region of interest (ROI) of the fingers and hand of a healthy control (A) and a CUrt subject (B), and corresponding time profiles (C and D).

We next analyzed the apparent shunting of blood flow away from the digits. A direct comparison of the spatial-temporal re-warming profile of the hand and all fingers shows a modest increase in deeper blood flow in a control (Figure 6A, C), whereas there is a divergent pattern in a patient (Figure 6B, D) who displays a rapid increase in hand but not finger temperature. Thus, blood flow alterations that follow mast cell degranulation are associated with pronounced disturbances in regional blood flow.

**Figure 7:**
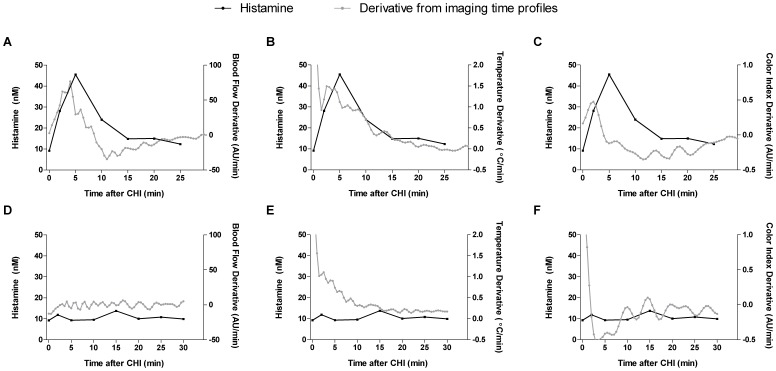
Association of histamine and imaging derivatives. Analysis of mean serum histamine levels for patients (A–C) and controls (D–F) plotted against the composite derivative (i.e. rate of change) of imaging time profiles for all subjects (see methods) for blood flow (A, D), temperature (B, E) and color index (C, F). For example, AU/min is a rate of change of AU with respect to time (dAU/dt). The data supports the association between histamine release as a surrogate marker for mast cell degranulation and vascular changes in those with CUrt, but not healthy subjects.

To further establish the relationship between mast cell degranulation and vascular changes, we compared peak histamine release in patients and healthy subjects to maximum rate of change of imaging signals (Figure 7). The maximum rate of increase in blood flow corresponds to the peak in time of maximal histamine release (Figure 7A). For temperature and color index derivatives, the peak at ∼2.5 min post challenge corresponds to the sharp rise of histamine levels in the patient group (Figure 7B, C). Correlations were not detected in controls (Figure 7D, E, F). A significant correlation was found between histamine levels and blood flow and temperature change from baseline but not color index (Figure S3). This analysis further confirms the association between histamine release as a surrogate marker of mast cell activation and vascular changes in those with CUrt.

### Antihistamines cause Decreased Dermal Blood Flow

**Figure 8:**
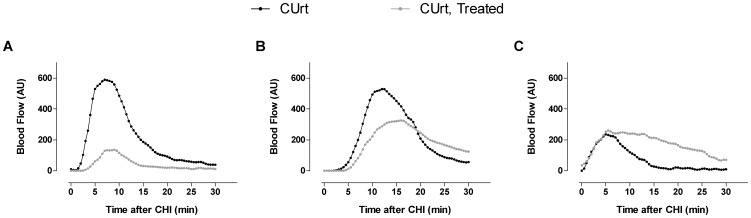
Blood flow profile for CUrt subjects treated with antihistamine. Three CUrt patients were re-imaged using LSCI during CHI while taking antihistamines (10 mg cetirizine). Blood flow time profiles for three patients (A, subject 1; B, subject 2; C, subject 4) before (black line) and after (gray line) treatment. The region of interest used for this plot included only the area between the base of the wrist and the knuckles.

Three patients were re-imaged during challenge testing while on a standard regimen of cetirizine (10 mg daily) for a minimum of one week. Blood flow signal decreased markedly in subject 1, partially in subject 2 and was unchanged in subject 4 (Figure 8), thus indicating that treatment with antihistamine restores normal blood flow in some patients. These findings correlated with the clinical response in each patient. The marked reduction in LSCI blood flow signal in subject 1 is shown pictorially in Figure S4. Thus, LSCI detected an objective reduction in superficial blood flow response, which corresponded with symptom relief. These data support the conclusion that documentation of changes in vascular blood flow offers a way to begin to understand the physiologic consequences of specific interventions on the vascular changes associated with provocation of physical urticarias in patients suffering from these disorders.

## Discussion

In this study we used real-time imaging technologies to better understand vascular changes associated mast cell degranulation within tissues. We found that the vascular response associated with mast cell degranulation is characterized by increased blood flow, thermal warming, vasodilation (Figure 3 and S2), and recruitment of collateral circulation (Figure 6). Treatment with antihistamine partially restores the normal pattern of superficial blood flow and is associated with less tissue edema (Figure 8 and S4). To associate these changes with mast cell activation, we documented a rise in serum histamine following CHI in affected subjects (Figure 1A) [12], [16], [17].

Histamine levels were elevated accordingly when patients were stratified based on severity (Figure 1C, S1). Serum tryptase levels remained unchanged (Figure 1D) although local release of tryptase by mast cells was documented (Figure 2). While it is known that tryptase is released by human mast cells together with histamine in vitro [13], there are discrepancies between the detection of these mediators in clinical disease [18], [19] attributed to the fact that mature tryptase occurs in complex with proteoglycan, which limits diffusion into the vascular compartment while it can be seen locally within the dermis associated with mast cells [20]. The lack of detection of serum tryptase despite a rise in histamine entertains the possible contribution of histamine from basophils. This is unlikely for the following reasons: First, tryptase is also found in human basophils although the amount may vary considerably [21]. Thus the absence of tryptase in blood could also be used as an argument against the involvement of blood born basophils in the cold induced reaction. Second, Kaplan et al., reported in an in vitro model of acquired cold urticaria that histamine was only induced in chilled skin tissues and not released from chilled blood leukocytes and purified basophils [22]. Third, the serum of patients with cold urticaria does not activate basophils [23]. Thus, these observations implicate mast cells as the primary source for the histamine-dependent reaction in patients with CUrt.

LSCI, IR and PLC simultaneously acquired images during CHI challenge. A pictorial comparison of the three modalities at baseline and 10 minutes post-CHI (Figure 3) displays a clear difference in dermal blood flow patterns and inflammatory response in patients versus controls. In patients, the marked inflammatory response is seen earlier in the hand than in the fingers (compare Figure 3B, to Figure S4B). We were able to characterize the onset, peak and rewarming of tissue in patients and to identify hemodynamic markers. Using LSCI post-CHI, patients displayed significantly higher maximal, more delayed onset and slower recovery of mean blood flow (Figure 4A). IR imaging detected a rapid onset and exaggerated temperature response above baseline (Figure 4B). Color indexes using PLC revealed differences in delayed time to reach maximal color and recovery (Figure 4C). These findings support the conclusion that patients with CUrt during cold exposure have an exaggerated response to natural body heat preservation.

We demonstrated in a subset of individuals that the warming pattern of the hand and fingers is homogeneous in comparison to a patient with CUrt where there is a more rapid and dramatic recovery of the palm versus the fingers (Figure 6). Given mast cell density is similar in superficial and deep dermis [20], the cooling of more tissue in the palm may lead to greater efflux of histamine and other vasodilator mediators and thus greater blood flow. At the other end of the spectrum, although the rewarming pattern following cold exposure in those with Raynaud’s syndrome is homogenous, it is much slower than controls due to dermal episodic ischemia [24]. Our observations provide experimental support to those patients reporting variable regional responses to cold exposure.

When correlating microvascular changes to histamine, we demonstrated that the highest rate of change in response correlates with the rise in histamine (Figure 7 and Video S1). Repeat analysis of three patients on cetirizine highlights the utility of imaging to quantify a decreased superficial inflammatory response to cold challenge following administration of an anti-allergic drug (Figures 8 and S4). The spectrum of responses is likely a consequence of the need to block other mast cell derived or induced mediators in patients that have more severe pathology.

Using LSCI which assesses superficial blood flow, IR which reflects changes in blood flow in superficial and subcutaneous tissues and PLC which detects the skin reddening caused by both moving and stagnant red cells in blood capillary loops close to dermal papillae, we were able to examine the sequence of events in a local inflammatory response that occurs at different vascular plexuses within the skin microvascular bed. We thus observed that the time of onset, max rate of change, and mean time to reach maximum for three imaging signals did not dissociate (Figure 7). This observation supports the conclusion that rewarming is associated with vasodilator recruitment of microvasculature in all vascular plexuses simultaneously. These vascular changes would be expected to facilitate the inflammatory response by increasing exposure of blood born cells and proinflammatory proteins to the area of insult.

Inducible inflammation in cold urticaria is a unique model that allows tissue mast cell dependent events to be studied in humans at baseline and during challenge testing. We were able to thus make a number of new and novel observations, which included the dermal phenotype of microvascular hypereactivity, time to recovery and response to antihistamine. It is tempting to speculate that patients with cold-induced urticaria are exhibiting an exaggerated protective response to cold whereby mast cell degranulation represents a protective mechanism to maintain blood flow and thus prevent more extensive tissue damage, much as has been suggested for cutaneous vasodilatation provoked by pressure [25].

## Materials and Methods

### Ethics Statement

Patients with CUrt and non-atopic healthy volunteers between 6 months and 65 years of age provided informed written consent under protocol 09-I-0126, approved by the Institutional Review Board (IRB)/Ethics Committee of the National Institute of Allergy and Infectious Diseases and adhered to Declaration of Helsinki Principles. Informed written consent approved by the IRB was completed by next of kin, legal caretakers or legal guardians on behalf of minors/children. In addition, minors/children completed and signed a minor patient assent to participate.

### Subjects and Demographics

The age range for patients (n = 7) with CUrt was 12–46 years (mean 28) (Table S1). The majority of patients were female (71%). The average duration of symptoms was 11.4 years, with an average severity of 9 out of a 15-point scale (Appendix S1) and an average CSTT threshold of 3.7 minutes. The majority of the patients were not dermatographic (14%), but were atopic (71%) with a total average serum IgE of 189 IU/mL and a resting basal average tryptase of 4.5 ng/mL. All patients had normal complement levels, negative cryoglobulins and undetectable cold agglutinin titers. All subjects were negative for cholinergic, exercise induced, pressure and local heat urticaria. The age range for control group (n = 6) was 12–56 years old (mean 39) (50% female).

On the initial evaluation and following a history and physical exam, participants underwent standard challenge testing for CUrt and the time for induction was established. Subjects were advised to withhold antihistamines, leukotriene antagonists, and other agents that could modify the induction of urticaria for 5 days prior to testing. Patients returned within 3 months for challenge testing with imaging and serial blood draws. Blood samples were stored and analyzed for mediators. After withholding all medications for 7 days, three of seven CUrt patients representing the spectrum of severity returned for additional challenge testing while on *cetirizine* (10 mg/D) for a minimum of one week. Four patients fell within the medium (6–10) and three within the high (11–15) severity groups. All controls had a zero severity score.

### Cold Stimulation Time Testing (CSTT)

To verify CUrt, all patients underwent CSTT using a 50 ml glass beaker of ice water (0°–2°C) placed on the volar surface of the forearm for 5 minutes, with observation of urticaria development upon rewarming for 30 minutes. As a marker of severity, the minimal time threshold to develop a singular circumscribed hive was established (Table S1).

### Imaging, Cold Hand Immersion (CHI), and Serial Blood Sampling

Prior to the challenge, an intravenous catheter was placed in the basilic vein of the left arm. Patients were acclimated to room temperature (22–24°C) and positioned for 15 minutes prior to testing. While sitting upright, the palm of the left hand was placed on a loosely strung tennis racket head adhered to a platform. The racket grid maintained the hand above the table surface, minimizing conductive heat exchange of the hand with the platform and allowing even evaporation of residual water.

One minute baseline imaging was followed by CHI with the hand submerged in cold water (10°C) to 2 inches above the wrist for 5 minutes [26]. After immersion, the hand was removed, dried and returned to the same position on the grid, whereupon imaging continued for 30 to 60 minutes. Serial blood draws were obtained at baseline and 0, 2, 5, 10, 15, 20, 25, 35, 60, and 120 minutes after immersion as shown in Figure S5.

### Histamine and Tryptase Assay

Blood samples were drawn into serum separation tubes, with serum aliquots stored at −80°C until analyzed. Serum histamine was measured using a competitive enzyme immunoassay (SPI-Bio, Bertin Pharma, France). Serum tryptase was measured by the ImmunoCAP 100 System (Phadia Inc., USA).

### Assessment of Vascular Response

Non-invasive optical imagers are able to measure microcirculatory events in the skin in real-time. These include laser speckle contrast imaging (LSCI), infrared thermal imaging (IR), and polarized light colorimetry (PLC). Such imaging provides quantitative and qualitative measurements of skin blood flow circulation, temperature and degrees of redness to correlate with clinical manifestations of skin disorders. LSCI is sensitive to microcirculatory perfusion to 1.0 mm of skin depth and has been used to evaluate perfusion dynamics in burns, wounds (laser Doppler) [27], diabetics [28], and cigarette smokers [29]. Skin temperature measured with IR reflects dermal metabolism and inflammation, providing a window into the physiologic status of the nervous and vascular systems. IR detects dynamic blood flow and pyrogenesis within 10 mm of skin depth and temperature gradients of 0.015**°**C. IR has been used to follow treatment responses to melanoma, [30] assess facial temperature in non-verbal individuals with severe motor impairment [31] and evaluate the delayed rewarming process in Raynaud’s phenomenon [24]. PLC is digital color photography that objectively measures epidermal skin color changes to 500 µm depth [32]. Absorption of red light in tissue remains relatively constant during challenge regardless of red cell concentration; however, absorption of green light increases as red cell concentration increases [33]. The color index calculation is proportional to the difference of red and green color bands, divided by the red color band, the latter of which is a normalizing factor to baseline skin color (i.e. melanin content). To interpret color index, higher values occur when absorption of green light increases, which corresponds with elevated relative red cell concentration.

LSCI, IR, and PLC cameras were positioned above the support platform and focused on the dorsal surface of the left hand during CUrt challenge testing.

### Imaging Instrumentation

The LSCI (Moor Instruments, UK) camera measures red blood cell flux (velocity × concentration) in living tissue. The camera was positioned 30 cm above the subject’s hand. Images were collected at 5 Hz, and each image has 113×152 pixels/frame and covers 15 cm × 20 cm. Required calibration was performed prior to the imaging session.

The IR camera (Santa Barbara FocalPlane, Lockheed Martin, USA) passively measures heat radiated from the skin within the wavelength range of 3–5 µm. Images (640×512 pixels/frame) were acquired at 2 Hz from a distance of 50 cm, covering an area of 28 cm×22 cm. To convert IR camera photon counts to temperature units, a multiple point calibration was implemented using blackbody standard (CI Systems SR-80, Israel) and a fifth-order polynomial was applied to calibration curve within temperature range between 28°C and 33°C.

The PLC device consists of a Canon 2Ti (Canon, Japan) camera equipped with a Macro-Ring Lite flash that quantifies changes in skin color, also visible to the eye. The flash and lens are both polarized and are aligned to be cross-polarized relative to each other. In this configuration, photons that reflect off the surface of the skin are filtered. Photons that penetrate deeper into the skin are depolarized and can pass through the lens filter. Images (5184×3456 pixels/frame) were acquired every 15 seconds at a distance of 35 cm, covering an area of 18 cm × 12 cm. The normalized difference between the red and green color bands was used to quantify color [33]. All three cameras were controlled remotely by separate computers and acquired images were displayed in real-time for visualization and saved for off-line processing.

### Dermal Imaging Analysis

To remove motion artifacts, images of each modality were aligned with a rigid-body transformation algorithm to a reference image using the edges of the hand as fiducial points. To analyze time profiles for blood flow, temperature and skin color, the same region of interest (ROI) for each modality was drawn for each subject, which included the area between the base of the wrist and the knuckles. Next, the time profile of each modality was calculated using the mean signal of the ROI of each image. In a subset of patients, this ROI was compared to ROIs on the fingers to follow the process of hand rewarming.

The mean of the one-minute baseline was subtracted from each point of the time profile to normalize patients. An elliptical low-pass smoothing filter (frequency cutoff of 0.01 Hz) was applied to each profile. These time profiles were then down-sampled to one point per 30 seconds for further analysis. Any residual motion artifacts in the profiles were removed manually by interpolation using a third-order polynomial. To investigate the rate of change, the first derivative of each down-sampled time profile was calculated as the difference between adjacent values divided by the 30-second time interval. The derivative results in imaging units per unit time. For example, AU/min is the rate of change in AU per minute for LSCI.

### Skin Tissue Immunohistochemistry

Skin biopsies were obtained at baseline and 15 minutes after CSTT (5 min) on the challenge site, placed into 10% neutral buffered formalin, embedded in paraffin and cut into 5-µm-thick sections. Local anesthesia was performed just before biopsy and did not delay the timing of the biopsy. Slides for immunohistochemistry were deparaffinized and stained using the Discovery XT and Red Map Kit from Ventana Medical Systems (VMS, Tucson, AZ, USA). The MAb to mast cell tryptase (aa1) (Abcam, Cambridge, MA, USA) was diluted in Antibody Dilution Buffer (VMS) 1∶100 and incubated for 2 hours. A biotinylated undiluted secondary antibody of Goat anti-Mouse SS Link, (Biogenex, San Ramon, CA, USA) was incubated for 32 minutes, followed with enzyme conjugate, and Fast Red chromogen.

### Statistical Analysis

Four parameters to characterize time profiles were calculated for each imaging modality, including baseline, maximum, time to maximum, and time to half-max post-max. Baseline was the mean of one-minute imaging prior to CHI. Maximum and time to maximum were the maximum signal after immersion and the time to that point. The time to half-max was the time at which the signal was equal to half of the maximum, (or for IR: equal to the average of the minimum and maximum) and is an estimate of recovery time. The correlation between clinical severity and threshold values and these parameters was calculated using Prism 5 (GraphPad, USA).

Mean group time profiles for each subject group were calculated. Two-way ANOVA analysis was conducted to test if average response curves were significantly different. Data were compared to a Gaussian distribution for verification that the data was normally distributed using the Shapiro-Wilk Normality Test. Data that were not normally distributed were analyzed using non-parametric tests. Statistical results of p≤0.05 were considered significant. Error bars are ±1 SEM.

## Supporting Information

Figure S1 **Correlation of histamine with disease severity.** Plot showing a significant correlation (p = 0.002) between serum histamine levels in all subjects at 10 min post CHI and disease severity.
(TIFF)

Figure S2 **Blood flow, temperature, and skin color images of a representative healthy control subject.** Showing lack of response to cold challenge in comparison to CUrt subjects (Figure 3). Images at baseline (A, C, E, and G) and at 10 minutes post CHI (B, D, F, and H). A and B, blood flow images by LSCI; C and D, temperature images by infrared (IR); E and F, skin color images by polarized light colorimetry (PLC); and G and H, visible light photography.
(TIFF)

Figure S3 **Correlation of histamine with vascular response.** Imaging values are compared to histamine levels collected at 10 minutes post CHI for LSCI (A), IR (B) and PLC (C). Significant correlation was determined in blood flow (p = 0.044) and temperature (p = 0.031) thus supporting the correlation between mast cell degranulation and the vascular response.
(TIFF)

Figure S4 **Blood flow image of severely affected CUrt subject treated with antihistamine.** LSCI images of subject 1 (Table S1) at baseline (A and C) and at 10 minutes (B and D) post-challenge, untreated and treated with cetirizine 10 mg daily. At baseline, no difference was observed in blood perfusion between treated and untreated. At 10 minutes after CHI, a decrease in blood perfusion was observed while on cetirizine.
(TIFF)

Figure S5 **Challenge Timeline: Sequence of blood sampling at defined time points that were collected during imaging.** Time sequence of blood draws, imaging, and CHI test. Each procedure was performed during the time specified by the shaded region. In some subjects, imaging was extended from 30 minutes to a maximum of 60 minutes.
(TIFF)

Table S1 **Patient Characteristics.** Includes patient demographics, severity rating, atopic status, baseline histamine, tryptase and total IgE. Seven patients performed the CHI test. Prior to CHI, a 15 point questionnaire was given to assess disease severity, where 1 is low and 15 is high on the 1 to 15 range scale (Appendix S1). Patients established a CSTT threshold time prior to CHI, which was the minimum time of cold exposure that induced a response.
(TIFF)

Video S1 **Histamine release and infrared temperature changes in sequence.** Real time infrared imaging of patient undergoing cold hand immersion shows rapid swelling and increase in temperature above baseline. Sequential release of histamine and corresponding IR profile of temperature sampled at a region of interested between base of the wrist and knuckles. Rise in histamine correlates temporally with maximal rate of change in thermal warming.
(MOV)

Appendix S1 **Physical Urticaria severity Index.** Questionnaire completed by all subjects to determine urticaria severity.
(PDF)
