# Supplementary figures and images for: Mast Cell Dependent Vascular Changes Associated with an Acute Response to Cold Immersion in Primary Contact Urticaria
Source: PLoS One. 2013 Feb 22;8(2):e56773. doi: 10.1371/journal.pone.0056773 (PMC3579929; doi:10.1371/journal.pone.0056773)

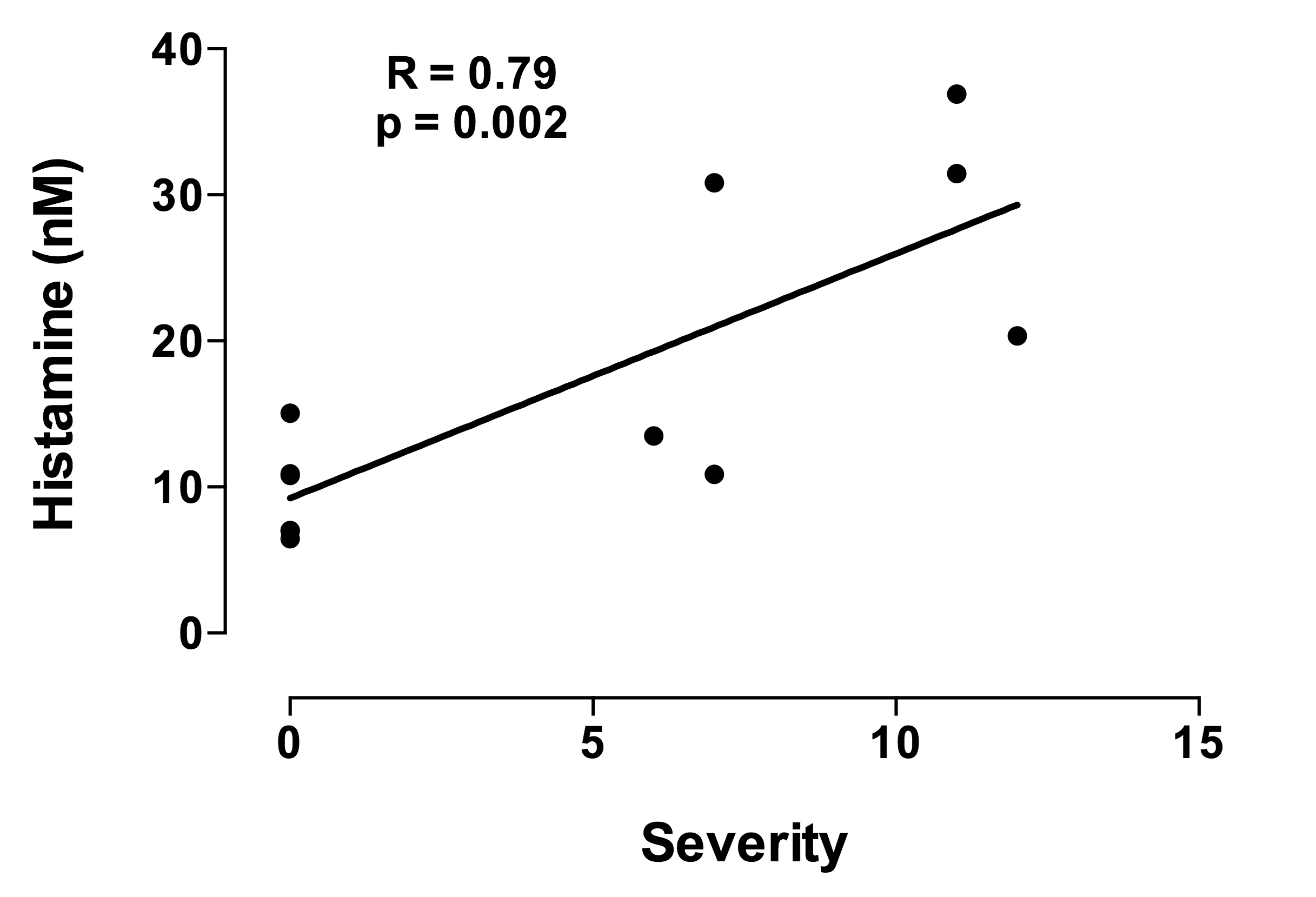

Supplement: Figure S1 — Correlation of histamine with disease severity. Plot showing a significant correlation (p = 0.002) between serum histamine levels in all subjects at 10 min post CHI and disease severity. (TIFF) [file pone.0056773.s001.tiff]

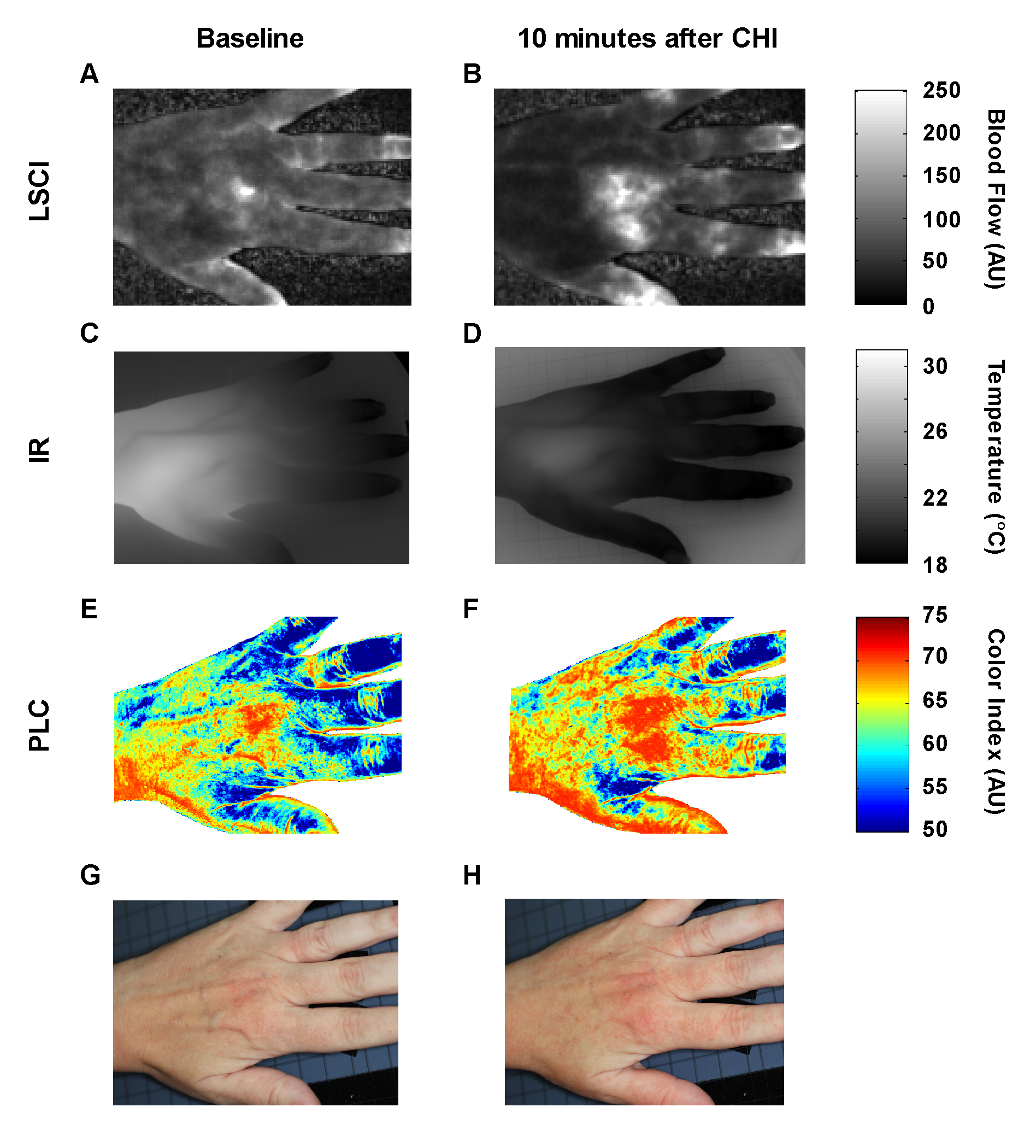

Supplement: Figure S2 — Blood flow, temperature, and skin color images of a representative healthy control subject. Showing lack of response to cold challenge in comparison to CUrt subjects (Figure 3). Images at baseline (A, C, E, and G) and at 10 minutes post CHI (B, D, F, and H). A and B, blood flow images by LSCI; C and D, temperature images by infrared (IR); E and F, skin color images by polarized light colorimetry (PLC); and G and H, visible light photography. (TIFF) [file pone.0056773.s002.tiff]

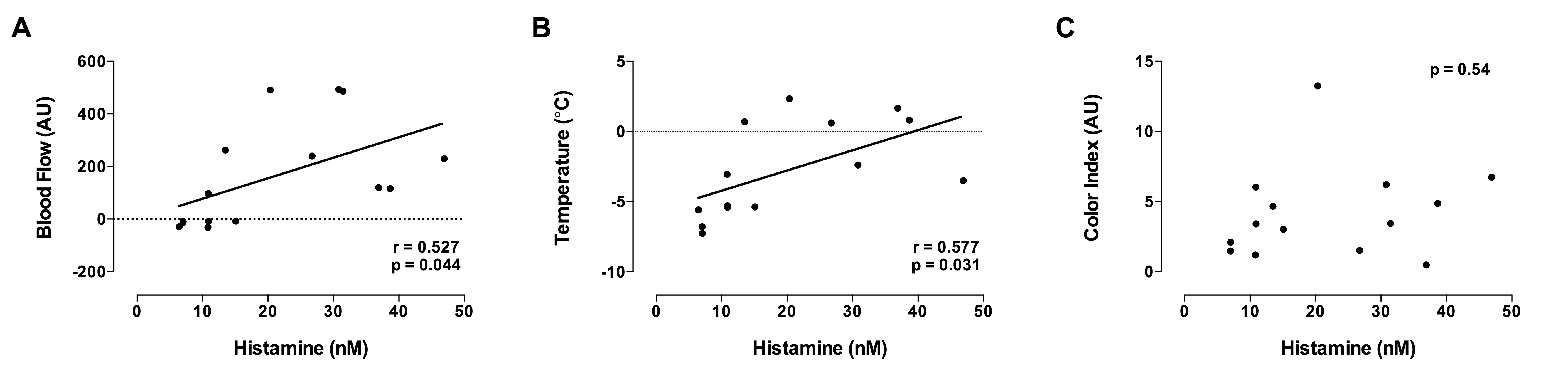

Supplement: Figure S3 — Correlation of histamine with vascular response. Imaging values are compared to histamine levels collected at 10 minutes post CHI for LSCI (A), IR (B) and PLC (C). Significant correlation was determined in blood flow (p = 0.044) and temperature (p = 0.031) thus supporting the correlation between mast cell degranulation and the vascular response. (TIFF) [file pone.0056773.s003.tiff]

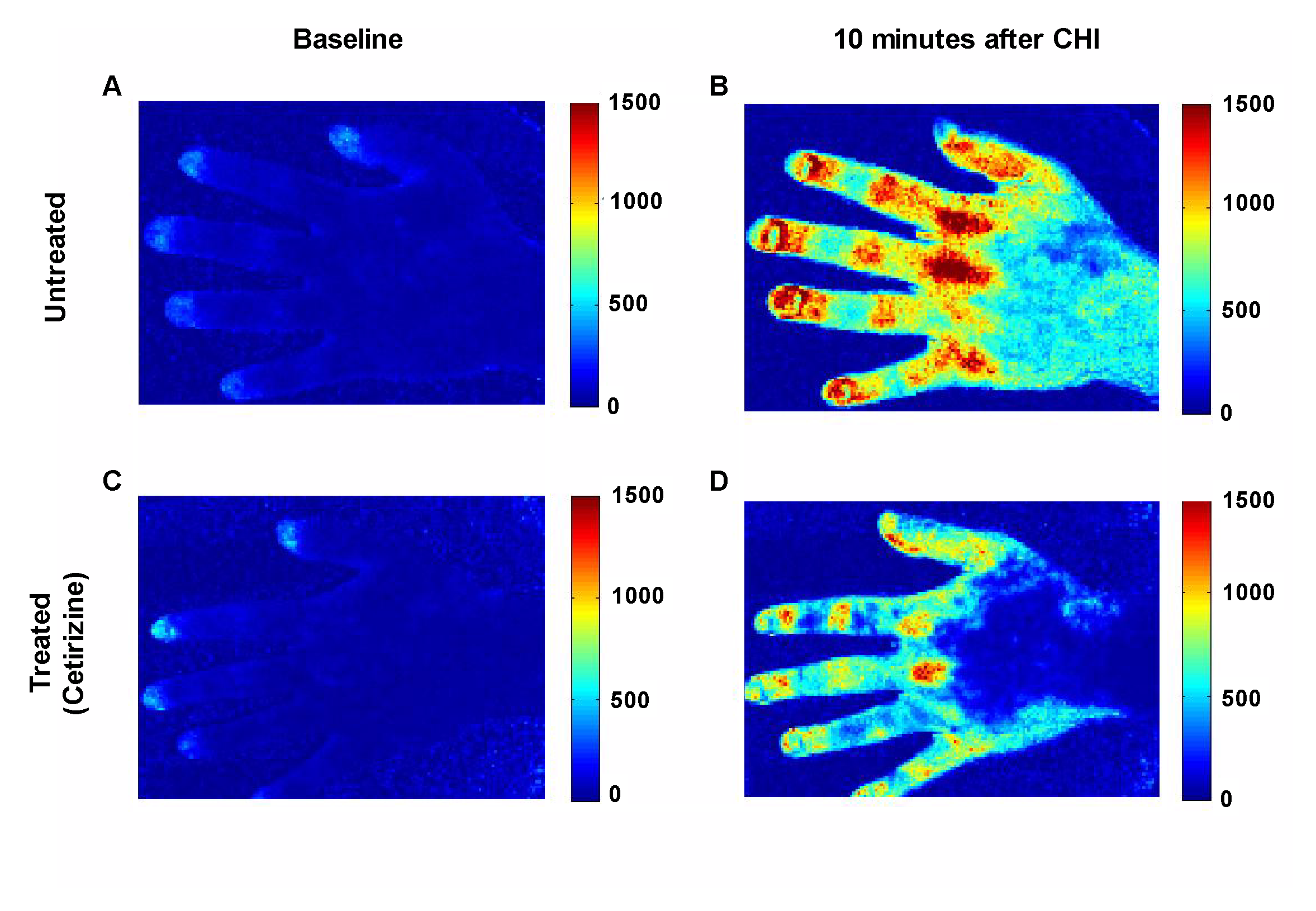

Supplement: Figure S4 — Blood flow image of severely affected CUrt subject treated with antihistamine. LSCI images of subject 1 (Table S1) at baseline (A and C) and at 10 minutes (B and D) post-challenge, untreated and treated with cetirizine 10 mg daily. At baseline, no difference was observed in blood perfusion between treated and untreated. At 10 minutes after CHI, a decrease in blood perfusion was observed while on cetirizine. (TIFF) [file pone.0056773.s004.tiff]

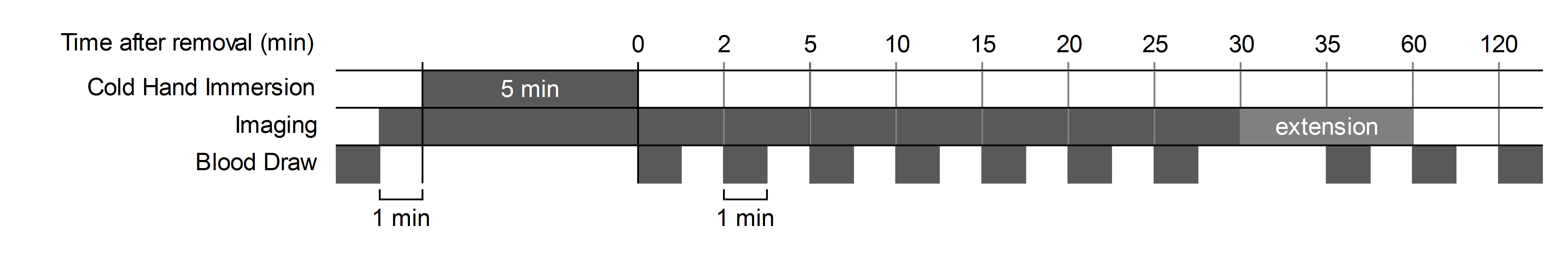

Supplement: Figure S5 — Challenge Timeline: Sequence of blood sampling at defined time points that were collected during imaging. Time sequence of blood draws, imaging, and CHI test. Each procedure was performed during the time specified by the shaded region. In some subjects, imaging was extended from 30 minutes to a maximum of 60 minutes. (TIFF) [file pone.0056773.s005.tiff]

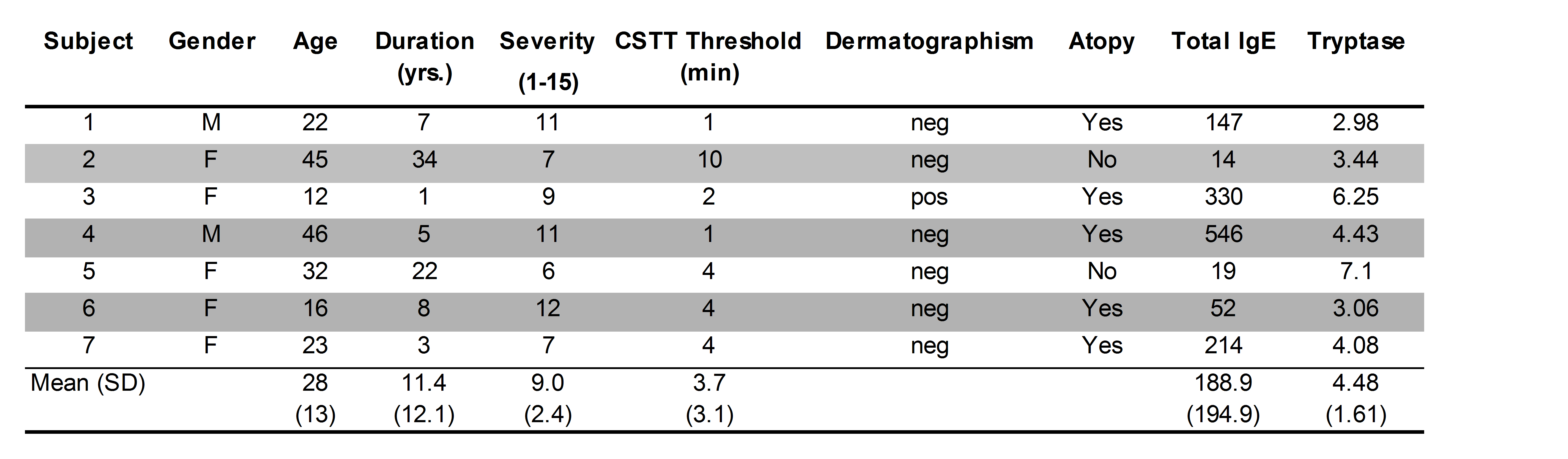

Supplement: Table S1 — Patient Characteristics. Includes patient demographics, severity rating, atopic status, baseline histamine, tryptase and total IgE. Seven patients performed the CHI test. Prior to CHI, a 15 point questionnaire was given to assess disease severity, where 1 is low and 15 is high on the 1 to 15 range scale (Appendix S1). Patients established a CSTT threshold time prior to CHI, which was the minimum time of cold exposure that induced a response. (TIFF) [file pone.0056773.s006.tiff]
