# Supplementary material for: Mast Cell Dependent Vascular Changes Associated with an Acute Response to Cold Immersion in Primary Contact Urticaria
Source: PLoS One. 2013 Feb 22;8(2):e56773. doi: 10.1371/journal.pone.0056773 (PMC3579929; doi:10.1371/journal.pone.0056773)
Supplement: Appendix S1 — Physical Urticaria severity Index. Questionnaire completed by all subjects to determine urticaria severity. (PDF) [file pone.0056773.s008.pdf]

Appendix S1. Physical Urticaria severity Index

Please read the following questions carefully and then mark your response with an √ in the ☐.

1. **What were the worst problems ever caused by your ___________________?** (*dermatographism, cold urticaria, delayed pressure urticaria, cholinergic urticaria, local heat urticaria, vibratory urticaria/angioedema, solar urticaria, aquagenic urticaria)*

☐ hives, redness or itching (1 point)
☐ deep swelling of the skin or mucous membranes / angioedema (e.g. eyes, lips, tongue) (2 points)
☐ circulatory complaints / dizziness or difficulty swallowing / difficulty breathing, nausea, vomiting, abdominal pain (3 points)
☐ unconsciousness / shock (4 points)

2. **When your urticaria is at it's worst: In which season did this occur**?

☐ Infrequent, no relationship to season (1 point)
☐ Winter only, ☐ Summer only, ☐ Spring only, ☐ Fall only (1 point)
☐ two or three seasons (2 points)
☐ During all seasons (means all year round)

3. **What is the maximum treatment that your urticaria has needed?**

☐ No treatment, I can avoid the trigger (1 point)
☐ Antihistamines, either on demand or used continuously as prevention (2 points)
☐ I needed to use my emergency medicine (EpiPen or hydrocortisone) then followed up for observation in the emergency department (3 points)
☐ I needed my emergency medicine and needed additional treatment by the emergency room physician (4 points)

4. **How often do you have hives?**

☐ I do not have urticaria; it is controlled by avoidance and / or medications
☐ rarely, about 1-2 times a month (1 point)
☐ Occasionally, about once a week (2 points)
☐ Often, several times a week (3 points)
☐ Daily or almost daily (4 points)
